# Editorial: Genetic Dissection of Important Traits in Aquaculture: Genome-Scale Tools Development, Trait Localization and Regulatory Mechanism Exploration

**DOI:** 10.3389/fgene.2020.00642

**Published:** 2020-06-25

**Authors:** Peng Xu, Lior David, Paulino Martínez, Gen Hua Yue

**Affiliations:** ^1^Fujian Key Laboratory of Genetics and Breeding of Marine Organisms, College of Ocean and Earth Sciences, Xiamen University, Xiamen, China; ^2^State Key Laboratory of Large Yellow Croaker Breeding, Ningde Fufa Fisheries Company Limited, Ningde, China; ^3^Department of Animal Sciences, RH Smith Faculty of Agriculture, Food and Environment, The Hebrew University of Jerusalem, Rehovot, Israel; ^4^Departamento de Xenética, Universidade de Santiago de Compostela, Lugo, Spain; ^5^Temasek Life Sciences Laboratory, National University of Singapore, Singapore, Singapore

**Keywords:** aquaculture, genomics, genetic breeding, GWAS, QTL, traits

After vigorous growth through the last decades, aquaculture industry reached a key milestone in 2014 when the aquaculture contribution to the supply of fish for human consumption overtook that of wild-caught fish for the first time. Fast developing aquaculture has achieved potential to feed 9.7 billion people by 2050 in a context of climate change, economic and financial uncertainty, and growing competition for natural resources (FAO, 2016). However, there are still many challenges for fast and sustainable development of aquaculture. These challenges include the generalization of genetically improved stocks, facing the climate change, environmental stressors, emerging pathogens and diseases, and improving of feed conversion rate, growth rate, and resilience. Genetic improvement and germplasm enhancement have been proved efficient and cost-effective approaches to take aquaculture production to the next level as needed.

Important progress has been made with genetic markers to support breeding schemes in the past decades, but now the flourishing applications of the next generation of genome sequencing technologies enables a further and bigger leap ahead. The genomes of more and more aquaculture species have been sequenced or are being sequenced, which facilitate the fast development of genome-scale technologies and tools. Genomic tools and resources combined with new more sophisticated bioinformatic tools are now available for many major aquaculture species, including reference genome sequences and their annotations, genome-wide polymorphic markers and genotyping platforms, high-density and high-resolution linkage maps, transcriptomic resources, and more recently, breakthrough techniques for understanding regulatory mechanisms underlying gene expression. Genomic scale fine mapping and genetic regulation of important performance traits, such as disease resistance, growth rate, sexual determination, and tolerance to various environmental stressors, have been studied for better understanding the regulatory mechanisms. Genome information supporting selective breeding programs have been initiated and are prepared to apply in many key aquaculture species.

It is our great pleasure that this Research Topic is presented to its readers. The present Research Topic focusing on genetic dissection of important traits in aquaculture collects 36 articles including two review articles and 34 original research articles from a total of 305 authors. The contributions cover diverse aquaculture species of finfish, shellfish, shrimps, crabs, and algae, representing the latest progress on genetic dissection of economically important traits with genome-scale tools and technologies. Herein we classified these articles into different topics to be highlighted.

Developing genome resources and genome-scale genetic tools constitute a fundamental step for genome-wide genetic analysis. In the past decade, reference genomes of aquaculture species have been quickly assembled linked to the advances in genome sequencing technologies and assembly algorithms as well as computation power enhancement. Four new reference genomes are reported in this issue, including a chromosome-level reference genome of Chinese Seabass (*Lateolabrax maculatus*) (Chen et al.) and three draft genomes of Sterlet sturgeon (*Acipenser ruthenus*) (Cheng et al.), Kanglang white minnow (*Anabarilius grahami*) (Jiang et al.), and a brown algae (*Saccharina japonica*) (Liu T. et al.). Linkage maps have been considered as the traditional genetic tool for trait dissection and construction of highly-dense chromosome-scale maps is addressed in several contributions. Three high-density linkage maps, for mud crab (*Scylla paramamosain*) (Waiho et al.), black tiger shrimp (*Penaeus monodon*) (Guo et al.), and channel catfish (*Ictalurus punctatus*) (Zhang S. et al.) respectively, were constructed based on Restriction Site associated DNA sequencing (RAD-seq or derivative methodologies). Sex- and growth-related traits are mapped in these three new linkage maps accordingly. The increasing genome resources and tools will facilitate genetic studies on important traits to tackle genomic selection in those aquaculture species.

Comparative transcriptomics is an effective approach to compare gene expression profiles between different samples that are challenged with different conditions or exhibit distinct phenotypes, which provides insights into molecular functions and biological processes underlying target traits. Comparative transcriptomics in tilapia, Chinese seabass, crucian carp (*Carassius carassius*), X-Ray tetra (*Pristella maxillaris*), tongue sole (*Cynoglossus semilaevis*), sea urchin (*Strongylocentrotus intermedius*), and Pacific oyster (*Crassostrea gigas*) treated at differential temperature and salinity or presenting significant differences in growth performance or ploidy are reported in this issue (Bian et al.; Hu et al.; Lin et al.; Liu J. et al.; Nitzan et al.; Tian et al.; Wang Q. et al.; Zhan et al.; Zhang F. et al.). Additionally, comparative genomics and phylogenetic analysis of immune-related genes aid at understanding of the structure and function of the immune system of Senegalese sole (*Solea senegalensis*) (García-Angulo et al.). Non-coding RNAs play important roles in transcriptome and translation regulation. Investigation on sncRNA, circRNA and miRNA was conducted in European sea bass (*Dicentrarchus labrax*) (Sarropoulou et al.) and turbot (*Scophthalmus maximus*) (Xiu, Jiang et al.), providing insights into the regulation mechanisms in these two species. DNA methylation, the most widely studied and most well-understood epigenetic modification, has been reported to play crucial roles in gene regulation processes, such as those related to sexual development. Piferrer et al. discuss the model of Conserved Epigenetic Regulation of Sex (CERS) and the use of CERS to make testable predictions on how sex is epigenetically controlled and to better understand sexual development primarily in fish. DNA methylation profiles in disease-resistant and disease-susceptible Chinese tongue sole against *Vibrio harveyi* infection are also compared and the results highlight that artificial selection for disease resistance may change methylation levels in important immune-related genes (Xiu, Shao et al.).

Many economically important traits in aquaculture are quantitative traits. Quantitative trait loci (QTL) mapping and genome-wide association studies (GWAS) are primary approaches to dissect the architecture of such traits, and to identify genomic regions, the underlying genes and eventually causative mutations that contribute to trait variation. Recent advances in high-throughput genotyping technologies facilitate more accurate trait dissection in many aquaculture species using GWAS and QTL mapping. Here we collect a number of articles under this topic, representing over one third of all contributions. Sex determination loci and candidate genes are identified in channel catfish (Zhang S. et al.) and black tiger shrimp (Guo et al.) via QTL mapping with high-density genetic linkage maps. Disease resistance is critical for aquaculture species, and resistance traits against sea lice, myxozoan parasite *Enteromyxum scophthalmi* and acute hepatopancreatic necrosis disease (AHPND) in Atlantic salmon (*Salmo salar*) (Robledo et al.), turbot (Ronza et al.), and Pacific white shrimp (*Litopenaeus vannamei*) (Wang, Zhang et al.) are respectively reported. GWAS on very diverse traits including growth, body shape, feed conversion ratio (FCR), filet quality, polyunsaturated fatty acids (PUFAs) content and glycogen content are also reported, indicating that this is a key and active research field in aquaculture (Ali et al.; Besson et al.; Kyriakis et al.; Liu S. et al.; Waiho et al.; Wang Q. et al.; Zhang H. et al.; Zhang Q. et al.; Vallejos-Vidal et al.).

Research communities in some aquaculture species have developed high density SNP genotyping arrays, which expedite population genomic studies and germplasm evaluation of those species. Barria et al. report their population genomic structure and genome-wide linkage disequilibrium analysis in three Chilean commercial populations of Atlantic salmon with different origins using a 159K SNP genotyping array. Xu et al. conduct a population genomic analysis to determine the genetic architecture of 2,198 individuals in 14 common carp populations worldwide using a 250K SNP genotyping array. Yoshida et al. genotype three farmed Nile tilapia (*Oreochromis niloticus*) populations in Latin America using a 50K SNP panel, and population genetic analysis revealed short-range LD decay for three populations.

Abundant genome resources, well-established genotyping technologies and well-characterized germplasms have boosted genome selection applications on the aquaculture species, as it is demonstrated in this issue by Palaiokostas et al. on KHV resistance breeding in common carp. Flourish applications of genome selection are expected on more and more farmed species in aquaculture industries in the foreseeable future. In addition to genome selection, Wang, Yang et al. highlight their findings on generating goldfish-like fish via interspecific hybridization of female koi carp × male blunt snout bream and indicate the potential to form new species.

Overall, advances of genomic technologies and their quick applications are accelerating genetic dissection of important traits in many aquaculture species. The better understanding of the genetic basis and gene regulations of economically important traits will further expedite genetic improvement using diverse approaches, and ultimately ensure the fast and sustainable growth of aquaculture industries globally.

We hope the aquaculture community will find this Research Topic to be an informative and useful collection of articles. As editors of this topic, we would like to thank the authors for their contribution to novel knowledge of this topic. We are grateful to all referees for their careful evaluation of the papers sent to them. Appreciation is also expressed to the numerous colleagues who responded to the call for papers, but whose interests could not be accommodated within the confines of this Research Topic. Finally, we glad to acknowledge Frontiers in Genetics for supporting this Research Topic.

## Author Contributions

PX prepared the draft editorial. LD, PM, and GY revised the manuscript. All authors contributed to the article and approved the submitted version.

## Conflict of Interest

PX was employed by the company Ningde Fufa Fisheries Company Limited. The remaining authors declare that the research was conducted in the absence of any commercial or financial relationships that could be construed as a potential conflict of interest.
